# A multispecies comparison of the metazoan 3'-processing downstream elements and the CstF-64 RNA recognition motif

**DOI:** 10.1186/1471-2164-7-55

**Published:** 2006-03-16

**Authors:** Jesse Salisbury, Keith W Hutchison, Joel H Graber

**Affiliations:** 1 Functional Genomics Program, The University of Maine, Orono, Maine 04469, USA; 2 The Jackson Laboratory, 600 Main Street, Bar Harbor, Maine 04609, USA; 3 Department of Biochemistry, Microbiology and Molecular Biology, The University of Maine, Orono, ME 04469, USA

## Abstract

**Background:**

The Cleavage Stimulation Factor (CstF) is a required protein complex for eukaryotic mRNA 3'-processing. CstF interacts with 3'-processing downstream elements (DSEs) through its 64-kDa subunit, CstF-64; however, the exact nature of this interaction has remained unclear. We used EST-to-genome alignments to identify and extract large sets of putative 3'-processing sites for mRNA from ten metazoan species, including *Homo sapiens, Canis familiaris, Rattus norvegicus, Mus musculus, Gallus gallus, Danio rerio, Takifugu rubripes, Drosophila melanogaster, Anopheles gambiae*, and *Caenorhabditis elegans*. In order to further delineate the details of the mRNA-protein interaction, we obtained and multiply aligned CstF-64 protein sequences from the same species.

**Results:**

We characterized the sequence content and specific positioning of putative DSEs across the range of organisms studied. Our analysis characterized the downstream element (DSE) as two distinct parts – a proximal UG-rich element and a distal U-rich element. We find that while the U-rich element is largely conserved in all of the organisms studied, the UG-rich element is not. Multiple alignment of the CstF-64 RNA recognition motif revealed that, while it is highly conserved throughout metazoans, we can identify amino acid changes that correlate with observed variation in the sequence content and positioning of the DSEs.

**Conclusion:**

Our analysis confirms the early reports of separate U- and UG-rich DSEs. The correlated variations in protein sequence and mRNA binding sequences provide novel insights into the interactions between the precursor mRNA and the 3'-processing machinery.

## Background

Cleavage and polyadenylation (3'-processing) are essential steps in eukaryotic mRNA formation that can effect transcript stability and function [1]. Processing of the 3'-end occurs on the nascent pre-mRNA as it is transcribed by RNA polymerase II [2]. Selection of the 3'-processing site is directed by interactions between the polyadenylation machinery and *cis*-acting elements found both upstream and downstream of the 3'-processing site. The principle upstream *cis*-acting element is the highly conserved AAUAAA hexamer, which interacts with Cleavage and Polyadenylation Specificity Factor (CPSF) and is found in the majority of metazoan transcripts [1,3]. Putative downstream elements (DSE) include the functional binding site(s) of the 64-kDa subunit of Cleavage Stimulation Factor (CstF) [4]. Interactions between CPSF and CstF, as well as polyA polymerase (PAP) and Cleavage Factors I and II (CFI and CFII respectively) are minimal essential requirements for *in vitro *polyadenylation [3].

### The DSE – one or two parts?

Unlike the upstream AAUAAA signal, whose description has remained largely unchanged since its discovery in 1976 [5], the DSE has had several descriptions. The DSE was initially characterized by conserved sequence patterns downstream of the 3'-processing site, resulting in estimated consensus sequences of UUUUCACUGC [6], GUGUUG [7], and CAYUG [8]. Two interesting early studies manipulated downstream sequences in test plasmids to produce a bipartite model of the DSE [9], consisting of a proximal UG-rich sequence and a distal U-rich element that act synergistically [10]. Further characterization of the DSE by deletion or substitution assays revealed UGUGUUGGAA [11], YGUGUUYY [12], AGGUUUUUU [13] and UUUUU [14,15] as elements actively involved with directing the polyadenylation event in specific transcripts and/or test systems. RNA binding assays indicated that CstF-64 interacts with UUUU with a spacing of 15–30 nucleotides downstream of the 3'-processing site [4].

The U-rich description was later challenged by SELEX binding assays performed on CstF-64 by two independent groups. Beyer *et al *used complete CstF complexes in cell extracts, and reported three distinct patterns: AUGCGUUCCUCGUCC, YGUGUYN_0–4_UUYAYUGYGU, and UUGYUN_0–4_AUUUACU(U/G)N_0–2_YCU [16]. Takagaki and Manley used a recombinant form of CstF-64 that included only the RNA recognition motif (RRM) and found preferred binding to a sequence that included both GU-rich (G(U)_2–4_G) and U-rich ((GU)_2–4_) components [17].

Statistical analysis of the DSE from information obtained from genomic alignments of *D. melanogaster *ESTs implicated the hexamers UGUUUU, UGUGUU and UUUUUU as DSEs [18]. Other studies involving genomic alignments of mammalian 3'-UTRs or ESTs reported only U-rich elements with no apparent consensus [19], a pentamer with at least 4 Us or 2GU/U [20], or the heptamer UGUGUGU [21]. An NMR solution of the vertebrate CstF-64 RRM structure was used to demonstrate binding to either (GU)_4 _or (GU)_4_UG, with a preference for the latter [22,23]. Through the wide variety of studies published to date, no clear consensus for the DSE has been demonstrated. In fact, the authors of the computational studies cited above argued against the existence of a single consensus. Review articles typically refer to a single UG-/U-rich DSE, in spite of the early evidence for two independent elements [9,10].

The present study was initiated to expand our understanding of the 3'-processing regulatory DSE sequences through a statistical survey that covers large sets of sequences across a broad phylogenetic range of metazoans. In addition, we also obtained and aligned multiple CstF-64 protein sequences for these same organisms, with the goal of identifying correlated changes in protein and probable nucleic acid binding sequences.

## Results

### Description of the datasets

**Figure 1:**
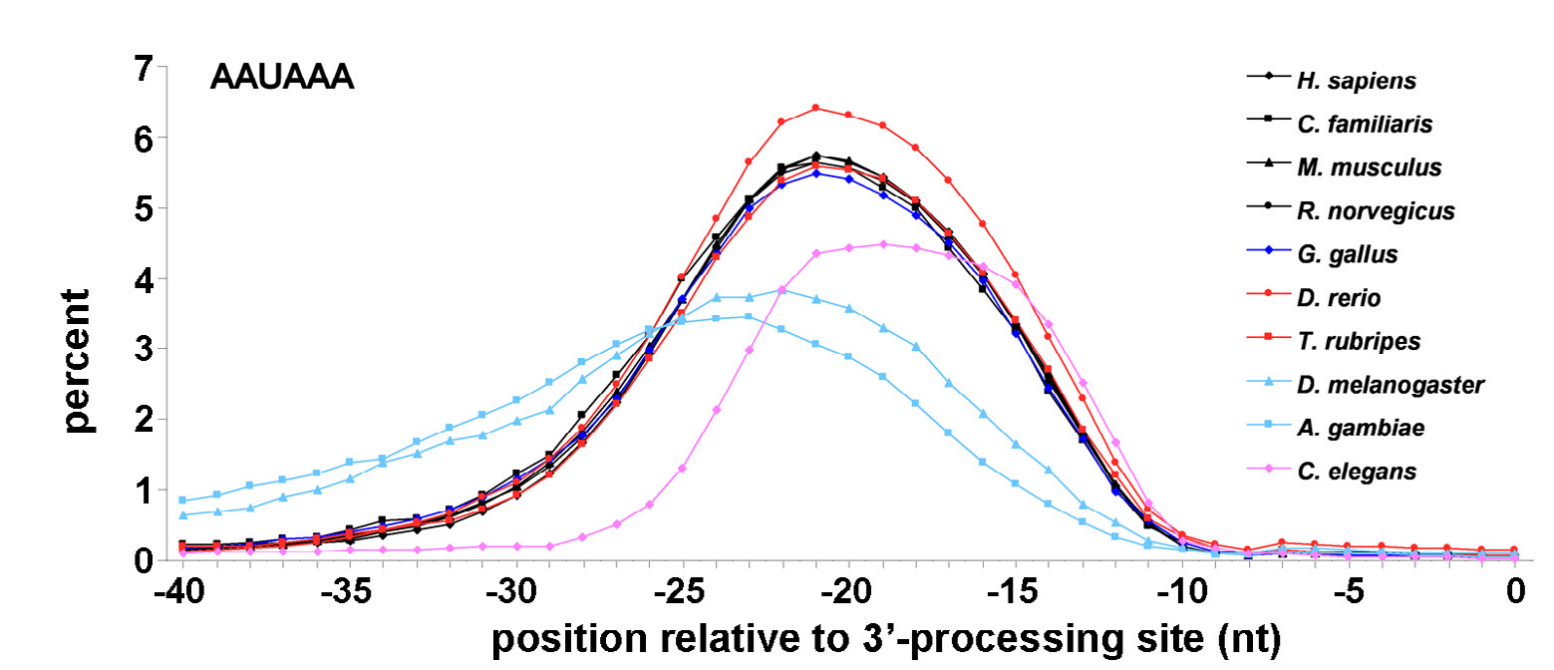
Positioning of the AAUAAA hexamer in the region upstream of the 3'-processing site for ten metazoan species.

We constructed a 3'-processing site sequence database (PACdb [24]) from 13,006,921 ESTs and 10 metazoan species including *Anopheles gambiae *(mosquito), *Caenorhabditis elegans *(nematode), *Canis familiaris *(dog), *Danio rerio *(zebrafish), *Drosophila melanogaster *(fruit fly), G*allus gallus *(chicken), *Homo sapiens *(human), *Mus musculus *(mouse), *Rattus norvegicus *(rat), and *Takifugu rubripes *(fugu). The numbers of non-redundant and high quality polyadenylation sites included in our analysis ranged from 902 in *D. melanogaster *to 10,060 in *H. sapiens *(Table 1). The quality of our training data was inferred from the presence of the well-documented canonical CPSF binding hexamer AAUAAA located up to 40 bases upstream of the processing site. The Positional Word Count (PWC, described in Methods) distribution for AAUAAA peaks at position -21 relative to the 3'-processing site in vertebrates and shifts to -23 in *A. gambiae*, -22 in *D. melanogaster *and -19 in *C. elegans *(Figure 1). Invertebrates demonstrated a reduced fidelity for the canonical hexamer compared to vertebrates, with the AAUAAA hexamer percentage falling markedly. The percentage of sequences with the AAUAAA hexamer is consistent with previous reports [20,25] and ranged from 69.9% to 77.9% for the vertebrates and 51.0% to 61.8% for the invertebrates (Table 1). We also tested for the presence of the most common variant, AUUAAA, and finally for any single base subsitution variant of the canonical hexamer (Table 1). Since the measured frequencies are in good agreement with previous results, we believe that the bulk of our sequences represent *bona fide *3'-processing sites.

### Positioning patterns of tetramers in the DSE region

**Figure 2:**
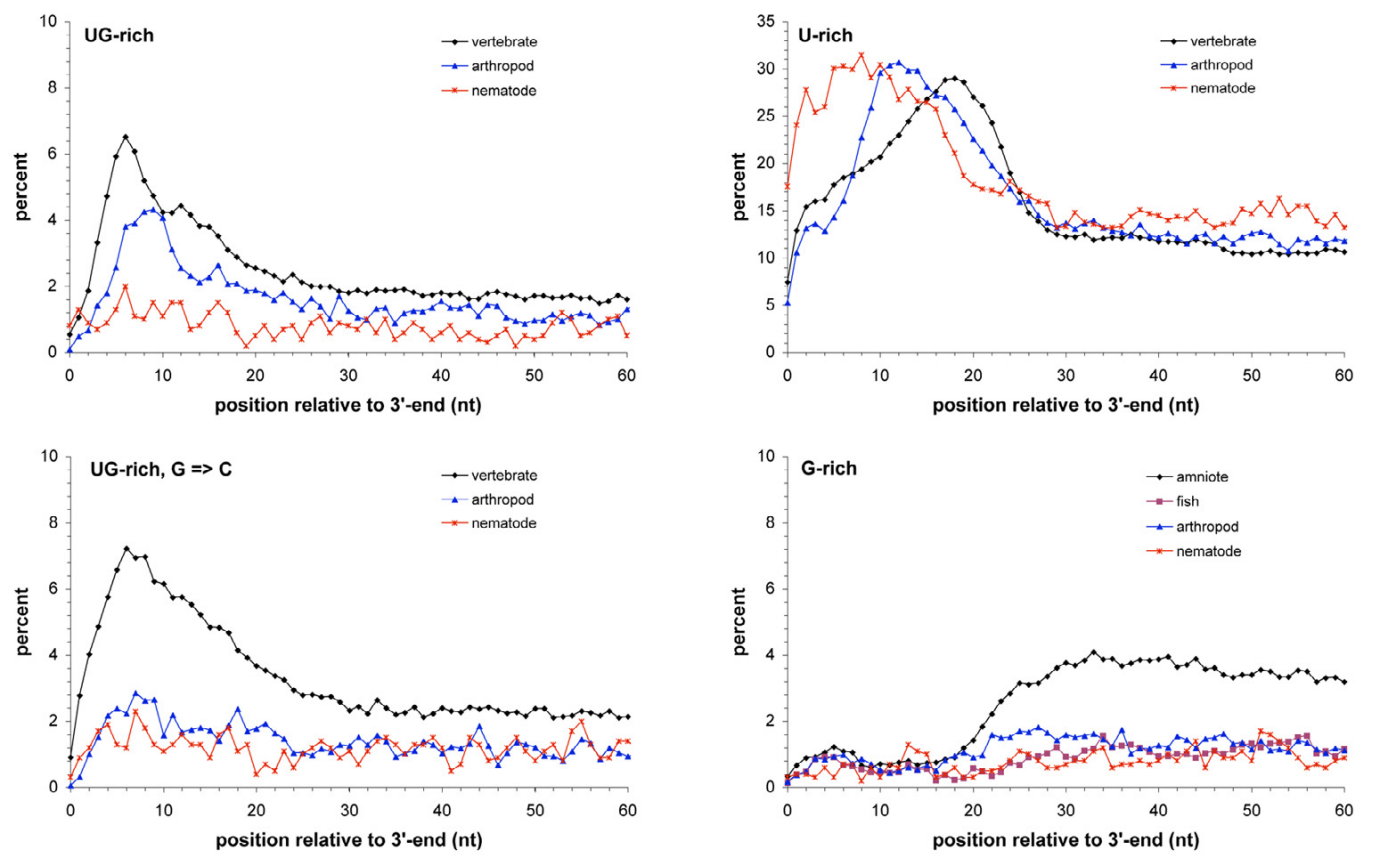
**Combined positional frequencies of selected groups of tetramers downstream of the 3'-processing site for ten metazoan species**. **A: **Proximal UG-rich element (including UGUG and GUGU), **B: **distal U-rich element (including all single base substitutions of UUUU), **C: **alternative proximal UG-rich element with G to C transversion (including UCUG, CUGU, UGUC, and GUCU) and **D: **G rich element (including GGGG, GGGA, GGAG, GAGG, and AGGG). In all panels, the vertical axis is the frequency of occurrence of any of the grouped tetramers at the position indicated along the horizontal axis.

Our initial PWC analysis of the DSE region was based on tetramers. While tetramers cannot unambiguously define the functional elements, they efficiently indicate positioning trends, as shown below. PWC probabilities for groups of tetramers with distinct, non-uniform positioning patterns are displayed in Figure 2. We display a subset of the tetramers, grouping words in the separate panels based on similar positioning and sequence content. The focus of the PWC analysis on positioning makes it immediately clear that there are at least three distinct patterns apparent downstream of the 3'-processing site.

The three patterns apparent in (Figure 2) can be approximately defined as (A) a UG-rich element positioned 5–10 nucleotides downstream of the 3'-processing sites (Figures 2A and 2C), (B) A U-rich element positioned 15–25 nucleotides downstream of the 3'-processing site (8–20 for *C. elegans*), and (C) a G-rich element positioned over 20 nucleotides downstream of the 3'-processing site. PWC results for all tetramers in the downstream region are available as a supplemental table [26]. The most robust pattern, in terms of frequency of occurrence across all sequences and organisms is the U-rich element (represented in Figure 2B by UUUU and all single base substitution variants), which has a strong positional bias in all species with maximum frequencies at positions 15 to 25 nt downstream of the processing site for the vertebrates and 8 to 15 for the invertebrates (Figure 2B.) The maximum positioning of the U-rich tetramers in *D. melanogaster *and *A. gambiae *are at positions 15 and 14 nt respectively and correlates with the 5' shift of the AAUAAA hexamer also seen in these species(Figure 1). U-rich tetramers in *C. elegans *cover a broadened range between 5 to 20 nt downstream of the processing site.

**Figure 3:**
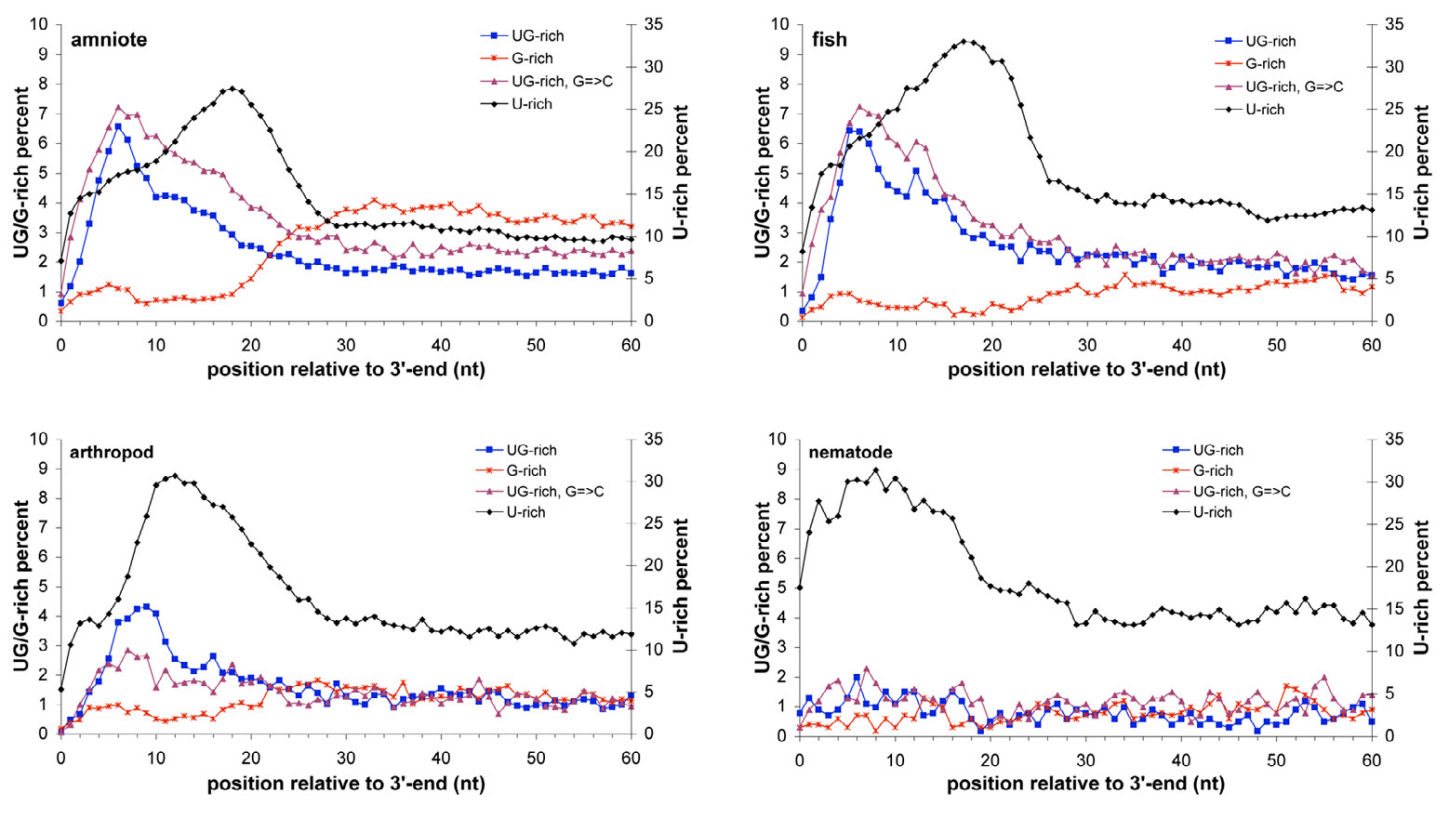
**Tetramer positioning patterns for specific classes of organism**. The plots from Figure 2 were grouped and averaged together according to the organism groupings listed as plot titles. In all plots, the U-rich element is plotted on the secondary vertical axis to allow greater detail to be observed in the other elements.

Examination of the data represented in Figures 2A, 2C, and 3 reveals significant variation in the UG-rich element between different species. For example, a comparison of Figures 2A and 2C indicates that variants of the UG-rich element with a G to C transversion (*e.g*., UCUG) have the same positioning indicating an acceptable functional substitution in all vertebrates, but not invertebrates. (A recent computational study of human 3'-processing sites also identified the UCUG-like elements [27].) *C. elegans *apparently does not have a UG-rich element based on the lack of significant positioning bias in either Figures 2A or 2C. It is also worth noting that both the positioning and the relative occurrence of the UG-rich element (without G to C transversion) changes in the arthropods compared to the vertebrates (Figures 2A and 3). The G-rich tetramers (represented by GGGG and all variants with a single G to A transition in Figures 2D and 3) appear to be a feature of only the amniote (represented here by mammals and *G. gallus*) 3'-processing sites.

### Delineating the DSE sequence content

While a tetramer-based PWC analysis clearly shows the positioning dependency of various sequence words, as noted above, it does not unambiguously describe the functional elements that produce the observed word distributions. Detailed delineation of RNA regulatory motifs is non-trivial, however, as while RNA elements are often defined by both sequence content and positioning, the standard computational pattern detection tools [28] typically consider only sequence content, with little or no weight given to positioning. Pattern recognition algorithms (*e.g*., MEME or the Gibbs Sampler) typically identify motifs as the patterns that most significantly stand out from the background implied by the surrounding sequence. One exception to this is the Improbizer [29], which models positioning according to a normal distribution.

While this is an improvement, examination of Figures 2 and 3 indicates that a normal distribution will only roughly approximate the observed positioning. We analyzed the 80 nucleotides downstream of our putative 3'-processing sites with a number of tools, including the Gibbs Recursive Sampler [30], MEME [31], the Improbizer [29], and a hexamer-based PWC analysis. Where necessary, we post-processed the results to include delineation of positioning distribution. We present the results of the Gibbs Sampler analysis here, whereas the other results are available in the online supplement [26].

**Figure 4:**
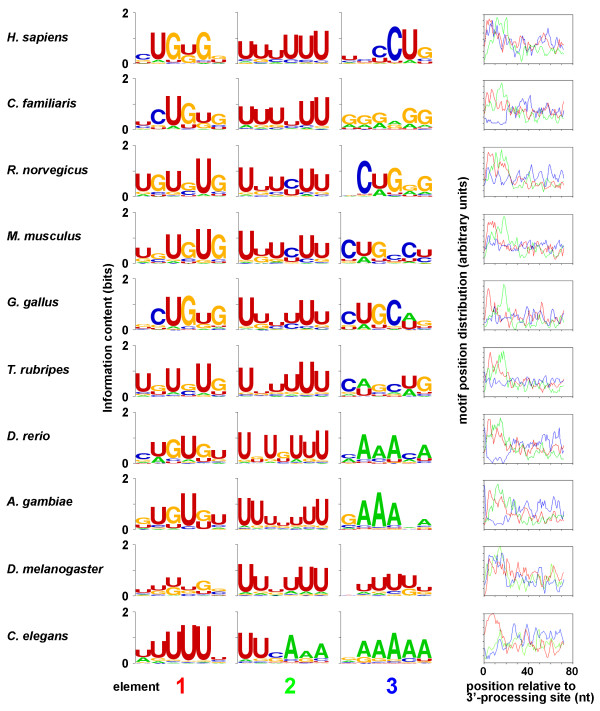
**Gibbs Recursive Sampler analysis of the DSE regions**. Typical results are shown from Gibbs Recursive Sampler [30] analysis of the organism-specific downstream sequence sets. The sequence content and positioning distribution of the motifs are represented by Sequence Logos [32] and line plots, respectively. The analysis allowed patterns to grow up to 10 nt in length, however the resulting patterns were consistently hexamers or heptamers, as shown. Positioning distributions were extracted from the output file and displayed graphically with a custom perl script. Elements 1, 2, and 3 are represented in the line plot as red, green, and blue lines, respectively. Full parameter lists for the analysis are described in Methods.

The Gibbs Sampler operates probabilistically, and can produce variable results upon repeated restarts. In addition, the large size of a number of our data sets (e.g., human, rat, and mouse) necessitated the selection of a random subset of the training sequences in order for the program to run in a reasonable time. A representative sampling of the Gibbs Sampler results is shown in Figure 4, using Sequence Logos [32] to represent of the sequence content, and line plots to represent the positioning distribution. These results are presented with the caveat that we specifically selected results that most closely reproduce the positioning patterns observed in the PWC analysis. Results from at least ten independent runs of each data set (with parameters as described in Methods) are available in our online supplement [26].

The Gibbs Sampler routinely identified the UG-rich element in all species except *C. elegans *(Figure 4), with positioning distributions often consistent with the patterns identified for UG-rich sequences in the PWC tetramer analysis (Figure 2). However, in several cases (*e.g*., elements 1 and 2 for *D. melanogaster *in Figure 4) the positioning distribution appeared to be a mixture of both the UG- and U-rich elements, likely indicating an overly "greedy" pattern description that encompassed both elements. Characterization of the U-rich element proved more elusive. Since our sequences are, in general, very U-rich, we could only identify U-rich motifs through either the use of a prior specification or by reducing the weight of the input sequence set in determining the background model. With these adjustments, we were able to characterize U-rich elements, such as those shown as motif 2 for nearly all organisms in Figure 4.

Consistent with the PWC tetramer analysis (Figure 2D), the Gibbs Sampler frequently identified far downstream G-rich motifs (*e.g*., motif 3 for *C. familiaris *in Figure 4), but only for amniotes. In contrast, the fish and arthropod far downstream regions produced an A-rich element, such as shown as motif 3 for *D. rerio, A. gambiae*, and *C. elegans*.

### Determining the DSE motif length

The Gibbs Sampler can vary the motif size (as can the Improbizer and MEME), selecting the length that produces the most statistically significant result. The Gibbs Sampler and Improbizer consistently returned motifs between 4 and 7 nucleotides in length, whereas MEME typically identified motifs between 12 and 15 nucleotides. Examination of the MEME results (available in the supplement [26]) revealed that the extended motifs resembled a concatenation of the UG- and U-rich elements that was dominated by either a strong UG-rich component in the first half or strong U-rich component in the second half. In addition, we also tested the fragmentation option of the Gibbs Sampler (data not shown), which allows the detection of non-contiguous patterns, under the constraint that the positioning between blocks must be fixed (or nearly so). Nearly all runs of all sequence sets resulted in contiguous motifs.

### Analysis of the CstF-64 RRM multiple alignment

**Figure 5:**
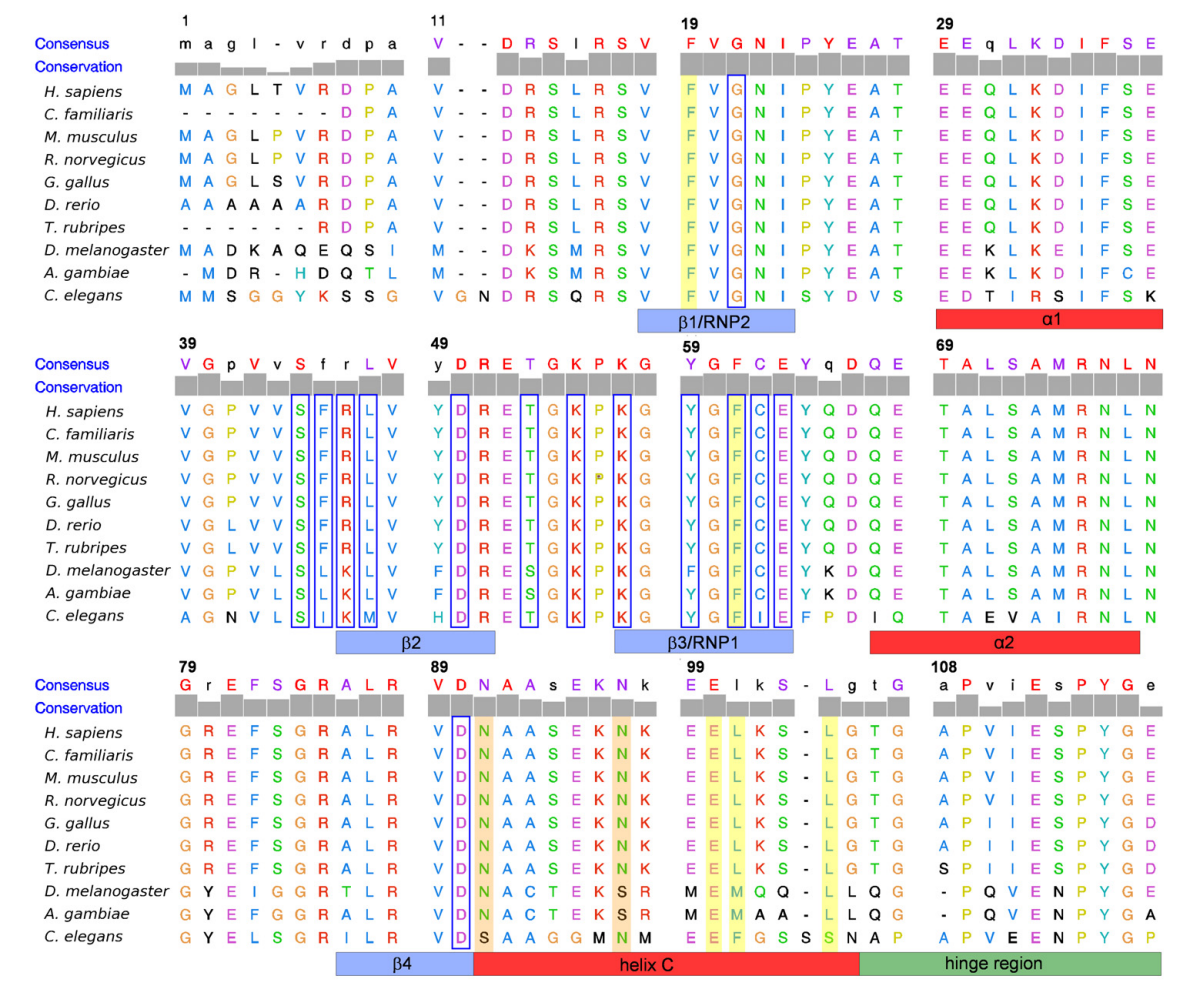
**Multiple alignment of CstF-64 N-terminal RRM and helix C region**. Colors: blue = *β*-strands; red = *α*-helix; green = hinge region (partial); yellow shaded = helix C stabilizing interactions [22]; boxed blue = residues most affected by RNA binding, according to NMR relaxation [23].

The CstF-64 RRM (or RNA binding domain) follows the well-conserved fold structure found in many other RNA-binding proteins (reviewed in [33]). Residues of *β*-strands one and three make up canonical motifs of RNP2 and RNP1 respectively, and are part of the larger RRM structure *β*_1_*α*_1_*β*_2_*β*_3_*α*_2_*β*_4 _(Figure 5). The vertebrate CstF-64 RRM is terminated by an additional *α*-helix (helix C) that lies across the *β*-sheet, occluding the projected RNA binding site [22]. The residues for the entire region spanning R_7 _through G_105 _(the end of helix C) are completely conserved in vertebrates, except for the single residue substitution of P_41 _→ L_41 _in fish. This near perfect conservation does not extend to invertebrates where numerous residue substitutions can be found. Across the same span of residues, percent identities between *A. gambiae, D. melanogaster*, and *C. elegans *and the vertebrate sequence are 72.7%, 66.7%, and 56.6%, respectively. (In addition, *C. elegans *has two insertions of 1 and 2 amino acids, respectively.) The substitutions are not uniformly distributed. If we restrict our analysis to only the *β*-sheet (highlighted in blue in Figure 5), the percent identities increase to 91.7%, 83.3%, and 79.1%, respectively. In contrast, the fifteen amino acids in helix C are much more variable, with percent identities of 33.3%, 33.3%, and 40%, respectively, and *C. elegans *also has a single amino acid insertion.

We further restricted our analysis to the amino acids previously identified as contributing to interactions between helix C and the *β*-sheet (F_19_, F_61_, N_91_, N_97_, E_100_, L_101_, and L_104 _[22]) or between the *β*-sheet and bound RNA (G_21_, S_44_, F_45_, R_46_, L_47_, D_50_, T_53_, K_55_, K_57_, Y_59_, F_61_, C_62_, E_63_, and D_90 _[23]). In Table 2, we list the subset for which changes are observed in the invertebrates.

While the percent identity of helix C is higher in *C. elegans *than for either *A. gambiae *or *D. melanogaster*, the substitutions in *C. elegans *are arguably more significant. Chou-Fasman *α*-helix and *β*-sheet propensities indicate that the S_94 _→ G_94_, E_95 _→ G_95 _and K_102 _→ G_102 _substitutions possibly prevent a stable helix C from forming in *C. elegans*. In addition, the vertebrate helix C includes three conserved lysines, all of which are oriented with their side chains pointing away from the *β*-sheet in the absence of bound RNA [22]. All of the lysines are replaced with non-charged residues in *C. elegans*, while *A. gambiae *and *D. melanogaster *have identical K_96_, a conservative K_98 _→ R_98 _substitution, but a non-charged substitution at residue 102.

### Extended multiple alignment

A multiple alignment of the complete sequences for the group of organisms analyzed is available as a supplement. The complete CstF-64 protein sequence consists of five distinct regions. The N-terminal (approximately 110 residues in vertebrates) CstF-64 RRM and helix C are highly conserved. In addition, the "hinge" region of CstF-64 (residues 110–210) which interacts with both CstF-77 and symplekin [34] is also highly conserved. The hinge region is followed by a low-complexity Proline-Glycine rich region (residues 210–410). The 12 contiguous MEAR(A/G) repeats (residues 410–470) including the interspersed RGG motifs, are weakly conserved outside of amniotes, if present at all. The remaining C-terminal residues (514–577) are highly conserved, reportedly reflecting the interaction between CstF-64 and the transcriptional coactivator PC4 [35].

## Discussion

### UG- and U-rich signals are distinct DSEs

Unlike the previous computational studies of the metazoan DSE cited above, our analysis explicitly includes characterization of the positioning biases relative to the 3'-processing site. While it is possible that the U/UG-rich DSEs comprise one motif, several aspects of our analysis lead us to the conclusion that our results are consistent with the presence of distinct UG- and U-rich elements as proposed by McDevitt *et al*. [9] and Gil and Proudfoot [10]. (This notably excludes *C. elegans*, which has no evidence of a UG-rich component.) While the positioning distributions of the U- and UG-rich sequences have considerable overlap, it is clear from Figures 2 and 3 that they are distinct. Previous experimental studies have occasionally produced longer putative elements that included both UG- and U-rich portions (*e.g*., [12] and [16]), however, if the functional element was a single longer element, we would expect UG- and U-rich positioning distributions with a common shape, but offset in position. In contrast, we observe distinct distributions that are more consistent with two independent elements separated by variable spacing. (It is worth noting that nothing in our analysis precludes these elements overlapping in an individual sequence.) Finally, the typical separation that we observe between the vertebrate UG- and U-rich elements (approximately 15 nucleotides between the UG-rich and U-rich positioning peaks in Figures 2 and 4) would imply a significantly longer RRM binding site than has been previously observed [33].

Our analysis indicates that the U-rich element is more prevalent than the UG-rich element in all species studied, and the sequence content and relative positioning of the U-rich element in all vertebrate species is consistent with previous *in vitro *polyadenylation assays [15] and CstF-64-pre-RNA UV cross linking studies [4], as well as the recent NMR studies of CstF-64 [22,23]. Somewhat paradoxically, the sequence content of the UG-rich element in the vertebrates is consistent with the results of the CstF-64 SELEX binding experiments [16,17]. Implications of these differences are discussed below. We believe that the historical difficulties in clear delineation of these elements are likely due to a convergence of several mitigating factors, including the degenerate sequence content of *both *the UG- and U-rich elements, significant overlap in both the positioning (Figures 2 and 4) and sequence content (Figure 4) of the two elements, and a typical emphasis on only sequence content in computational investigations.

### An interaction model for the CstF-64 RRM with precursor mRNA

Our comparative studies of the variation in the primary protein sequence of the CstF-64 RRM have highlighted differences in potentially critical residues that correlate with changes in the apparent binding sites identified by our statistical analysis. These correlations put us in a position to speculate on the mechanism of interaction between the RRM of CstF-64 and the downstream region of the precursor mRNAs. We hypothesize that the data presented here, in conjunction with previous published work, supports a model in which the proximal UG-rich element is involved in the necessary displacement of helix C [22] that exposes the *β*-sheet for binding to the distal U-rich element.

### Evidence for an interaction between the *β*-sheet and the U-rich element

UV-crosslinking of CstF-64 [4] revealed sequence content and positioning very similar to the U-rich element we describe in Figures 2 and 4. The NMR structures of the vertebrate CstF-64 RRM indicate that the binding pocket targets the UU di-nucleotide and that the larger UG di-nucleotide is discriminated against based on size [22]. Using specific oligomers, it was also shown that the (GU)_4 _sequence has a two-fold weaker interaction with the CstF than does the (GU)_4_UG sequence [23]. U-rich DSEs are a ubiquitous pattern of all organisms studied here, including *C. elegans*. The beta strands that form the RNA-binding sheet are also nearly perfectly conserved. The observed changes (F_45 _→ L_45 _in *D. melanogaster *and *A. gambiae*, F_45 _→ I_45 _in *C. elegans*, C_62 _→ I_62 _in *C. elegans*, A_86 _→ T_86 _in *D. melanogaster*, A_86 _→ I_86 _in *C. elegans*) are either conservative in side-chain substitution or oriented such that the side-chains face away from the binding pocket surface. In contrast, helix C displays considerable variation, as described below.

### Evidence for an interaction between helix C and the UG-rich element

The NMR studies showed that in the absence of RNA, helix C is stably bound to residues in the RRM binding pocket, thereby occluding it [22]. The residues responsible for this interaction are absolutely conserved in the vertebrate sequences we have examined. Significant changes found in the invertebrates likely disrupt or weaken the *β*-sheet to helix C interaction. These differences correlate with changes in the statistical patterns we identified for DSEs. Specifically, in *C. elegans*, ten of the sixteen helix C residues are changed from the vertebrate consensus, and many of these differences are non-conservative (Table 2). This correlates with complete absence of the proximal UG-rich element. In *D. melanogaster *and *A. gambiae*, the change in apparent affinity (Figures 2, 3, and 4) is more subtle, *e.g*., the UCUG-like variant of the vertebrate UG-rich element is absent. Several significant changes in helix C residues can be correlated with this change, including an N_97 _→ S_97 _substitution that could disrupt the hydrogen bonding to N_91 _predicted in the vertebrate structure. In addition, both *D. melanogaster *and *A. gambiae *are missing K_104_, which is conserved in vertebrates. According to the NMR structure [22], the K_104 _side chain is directed away from the beta sheet, an orientation that would make possible interactions between the charged amino side-chain and the RNA backbone. The observed correlations in protein sequence and apparent RNA affinity imply that helix C plays an important role in defining the DSE region. The importance of helix C is consistent with a forthcoming study of the in vitro binding affinities of variant forms of recombinant CstF-64 (R. Monarez, C.C. MacDonald, *pers. comm*.).

### What is the state of helix C during transcription?

The conformation of helix C during transcription is currently unknown, however it must be unwound or displaced prior to RNA binding [22]. If helix C was already displaced during transcription, the RNA binding process would be a single event. Although a single step binding process cannot be ruled out at this time, we find it to be unlikely as it would fail to explain the presence of both proximal UG-rich and distal U-rich elements. We speculate that helix C is structurally intact while scanning the nascent RNA and that a preliminary interaction is required for displacement. As described above, differences in helix C primary sequence correlate with changes in the pattern, or even existence, of the proximal UG-rich element. In addition, assuming a 5'-to-3' processivity, the proximal positioning of the UG-rich element is consistent with a role in the displacement of helix C that exposes the beta sheet for binding to the more prevalent distal U-rich element. If the model we propose is accurate, and CstF-64 is responsible for interactions with both DSEs, it provides an explanation for the discrepancy between sequence preferences observed in SELEX [16,17] and cross-linking studies. It remains an open question *why *SELEX measurements would favor the initial interaction with the UG-rich element.

### *C. elegans*, polycistronic transcripts and the proximal UG-rich element

The distinct changes in both the CstF-64 RRM sequence and apparent binding affinity in *C. elegans *are not surprising, given the known differences in 3'-processing. Approximately 15% of the genes in *C. elegans *are expressed in polycistronic transcripts [36], which are processed into monocistronic transcripts in a reaction that includes both 3'-processing and trans-splicing of a leader RNA to the downstream portion of the precursor [37]. Despite the peculiar nature of these transcripts, they are not sufficient by themselves to explain the absence of the proximal UG-rich element. The loss of the proximal UG-rich element is a transcriptome wide change and therefore likely reflects the RNA binding properties of the *C. elegans *CstF-64.

### The MEAR(A/G) repeats are not critical for UG- or U-rich DSE interactions

Previous reports speculated on a role for the MEAR(A/G) repeats in the CstF-64 pre-mRNA interaction [16]. Our analyses counter-indicate direct involvement of the MEAR(A/G) repeats in recognition of either the UG- or U-rich DSEs, since the MEAR(A/G) repeats are greatly reduced or absent in fish species (see the supplement), but the UG- and U-rich patterns are essentially unchanged from other vertebrates (Figures 2 and 3). In addition, SELEX studies that included only the N-terminal region of CstF-64 (approximately 130 residues) resulted in both UG- and U-rich binding patterns [17]. Although the function of the MEAR(A/G) like repeats is currently unknown, the striking reduction in fish and the invertebrates correlates with the loss of the G-rich signal in the far (greater than 20 nt) downstream region (Figure 2D). G-rich elements have been implicated as auxiliary 3'-processing elements, interacting with heteronuclear RNP complexes [38-41], acting as transcriptional pause sites [42], or forming a secondary structure based on the presence of G-quadruplexes [20].

## Conclusion

In our analysis of 39,578 high-quality 3'-processing sites spanning 10 genomes, we present the U/UG-rich DSE as two parts: a proximal UG-rich element with approximate positioning 5 to 10 nt downstream of the processing site and a distal U-rich element 15 to 25 nt from the processing site. Our results indicate that historical difficulties in classifying these elements are likely a consequence of their similarity in both sequence content and positioning. The distinct nature and positioning of the DSEs leads us to consider a model where the CstF-64 RRM interacts with both the UG- and U-rich elements separately and sequentially. Specifically, we hypothesize that the proximal UG-rich element contributes to the displacement of helix C, which exposes the RRM beta-sheet for subsequent binding to the distal U-rich element. While this model is speculative, it is consistent with both our results and previous studies.

Confirmation of this model through site directed mutagenesis or other techniques may lead to a better understanding of how the DSE region directs cleavage site choice and ultimately its role in alternate polyadenylation.

## Methods

### Extraction of polyadenylation sites

Previously we constructed a 3'-processing site sequence database (PACdb [24]) from 13,006,921 ESTs and 10 species (*A. gambiae, C. elegans, C. familiaris, D. rerio, D. melanogaster, G. gallus, H. sapiens, M. musculus, R. norvegicus*, and *T. rubripes*. The number of EST sequences available for each species ranged between 25,850 for *T. rubripes *and 6,002,331 for *H. sapiens *(see supplement). ESTs that mapped to a genomic location were scored by our discriminant function for polyadenylation evidence (described below). The number of total EST alignments that passed our discriminant thresholds were variable and ranged from 2,301 in *C. elegans *to 514,894 in *H. sapiens*. The polyadenylation sites implied by these ESTs were further grouped into unique genomic locations (± 25 nt) to account for sloppy polyadenylation and/or sequence data. Condensing the EST data in this manner reduced the number of implied 3'-processing sites to a range extending from a low of 1,003 in *C. elegans *to a high of 55,828 in *H. sapiens*. The average number of supporting ESTs at each unique genomic 3'-processing varied significantly between different organisms, introducing a bias that, if uncorrected, significantly increases the statistical weighting of rare sites in the more heavily sampled transcriptomes, *e.g., M. musculus *and *H. sapiens*. To correct for this bias, we used the mean number of supporting ESTs per unique 3'-processing site in each species as a minimum EST threshold. The final number of polyadenylation sites included in our analysis after applying redundancy thresholds ranged from 902 in *D. melanogaster *to 10,060 in *H. sapiens *(Table 1).

### PolyA discriminant function

Characterization of degenerate regulatory sequences is critically dependent on a high quality training set, therefore we developed a discriminant function that selects putative 3'-processing sites with both strong evidence of polyA tails on the EST, and absence of genomic A-rich regions that could signal a mispriming of the polyT primer used to generate cDNA clones. Internal priming is often addressed by setting a cutoff for the number of adenosine bases allowable in the genomic sequence flanking the putative 3'-processing site; this is a good estimate of primer stability, however, it does not take into account positional effects of mismatches. Since cDNA generation involves an enzyme binding/initiation step, mismatches to the 3' end of the polyT primer are critical and should be included in the scoring function. We modeled the combined effect of primer thermostability and reverse transcriptase processivity by incorporating an exponential weighting (according to position) into our function. We extracted 20 nucleotides downstream of all putative 3'-processing sites, from both the EST and genomic sequence. EST sequence was scored for likelihood of being a true 3'-end, whereas genomic sequence was scored for likelihood of false priming. Each sequence position (*x*) relative to the 3' processing site was scored independently (position score, eq. 1).

Position score (x)=1−∑1x1βe−x/β     (1)
 MathType@MTEF@5@5@+=feaafiart1ev1aaatCvAUfKttLearuWrP9MDH5MBPbIqV92AaeXatLxBI9gBaebbnrfifHhDYfgasaacH8akY=wiFfYdH8Gipec8Eeeu0xXdbba9frFj0=OqFfea0dXdd9vqai=hGuQ8kuc9pgc9s8qqaq=dirpe0xb9q8qiLsFr0=vr0=vr0dc8meaabaqaciaacaGaaeqabaqabeGadaaakeaacqqGqbaucqqGVbWBcqqGZbWCcqqGPbqAcqqG0baDcqqGPbqAcqqGVbWBcqqGUbGBcqqGGaaicqqGZbWCcqqGJbWycqqGVbWBcqqGYbGCcqqGLbqzcqqGGaaicqGGOaakcqWG4baEcqGGPaqkcqGH9aqpcqaIXaqmcqGHsisldaaeWbqaamaalaaabaGaeGymaedabaacciGae8NSdigaaiabdwgaLnaaCaaaleqabaGaeyOeI0IaemiEaGNaei4la8Iae8NSdigaaaqaaiabigdaXaqaaiabdIha4bqdcqGHris5aOGaaCzcaiaaxMaadaqadaqaaiabigdaXaGaayjkaiaawMcaaaaa@577C@

The scale parameter *β *was estimated to be 12 and reflects our estimate of the average number of A nucleotides involved in reverse transcriptase priming event. These position scores were summed to our total score if the base at position (*x*) = A:

total score=∑1≤20{position score (x)[I(Sx=A)]}     (2)
 MathType@MTEF@5@5@+=feaafiart1ev1aaatCvAUfKttLearuWrP9MDH5MBPbIqV92AaeXatLxBI9gBaebbnrfifHhDYfgasaacH8akY=wiFfYdH8Gipec8Eeeu0xXdbba9frFj0=OqFfea0dXdd9vqai=hGuQ8kuc9pgc9s8qqaq=dirpe0xb9q8qiLsFr0=vr0=vr0dc8meaabaqaciaacaGaaeqabaqabeGadaaakeaacqqG0baDcqqGVbWBcqqG0baDcqqGHbqycqqGSbaBcqqGGaaicqqGZbWCcqqGJbWycqqGVbWBcqqGYbGCcqqGLbqzcqGH9aqpdaaeWbqaaiabcUha7jabbchaWjabb+gaVjabbohaZjabbMgaPjabbsha0jabbMgaPjabb+gaVjabb6gaUjabbccaGiabbohaZjabbogaJjabb+gaVjabbkhaYjabbwgaLjabbccaGiabcIcaOiabdIha4jabcMcaPiabcUfaBjabdMeajjabcIcaOiabdofatnaaBaaaleaacqWG4baEaeqaaOGaeyypa0JaemyqaeKaeiykaKIaeiyxa0LaeiyFa0haleaacqaIXaqmaeaacqGHKjYOcqaIYaGmcqaIWaama0GaeyyeIuoakiaaxMaacaWLjaWaaeWaaeaacqaIYaGmaiaawIcacaGLPaaaaaa@6AC1@

where *I *is an indicator variable, equal to 1 if the argument is true, and 0 otherwise. Total score outputs of our discriminant function ranged from 0 (no As) to 9.7718 (all As). Thresholds used for high quality 3'-processing sites (see supplement) required a minimum total score of 5.5 for polyadenylation (EST sequence) and a maximum score of 4.5 (genomic sequence) for internal priming.

### Positional Word Count Analysis

Previous studies have shown that mRNA regulatory sequences can be characterized not only by sequence content, but also by relative positioning to a functional site, such as the 3'-processing site [18,20,21,27]. Our principal method is positional word counting (PWC), in which all sequence words of a given length (tetramers and hexamers in this work) are counted, recording both the occurrence and the position with respect to the 3'-processing site. When normalized, PWC results in a frequency for each *k*-mer at each position and can be interpreted as a probability of occurrence, conditional on the occurrence of a 3'-processing site at position 0. Putative functional sequences are identified as *k*-mers with statistically significant non-uniform positioning with respect to the 3'-processing site. We interpret different *k*-mers with similar positioning as evidence of acceptable substitutions in the functional element. Positional word frequencies (*pwf*) were calculated as the fraction of sequences with word (*w*) at position (*i*) (eq. 3).

pwfi=∑winwhere wi=word at position iand n=number of sequences     (3)
 MathType@MTEF@5@5@+=feaafiart1ev1aaatCvAUfKttLearuWrP9MDH5MBPbIqV92AaeXatLxBI9gBaebbnrfifHhDYfgasaacH8akY=wiFfYdH8Gipec8Eeeu0xXdbba9frFj0=OqFfea0dXdd9vqai=hGuQ8kuc9pgc9s8qqaq=dirpe0xb9q8qiLsFr0=vr0=vr0dc8meaabaqaciaacaGaaeqabaqabeGadaaakeaafaqabeWabaaabaGaemiCaaNaem4DaCNaemOzay2aaSbaaSqaaiabdMgaPbqabaGccqGH9aqpdaWcaaqaamaaqaeabaGaem4DaC3aaSbaaSqaaiabdMgaPbqabaaabeqab0GaeyyeIuoaaOqaaiabd6gaUbaaaeaacqqG3bWDcqqGObaAcqqGLbqzcqqGYbGCcqqGLbqzcqqGGaaicqWG3bWDdaWgaaWcbaGaemyAaKgabeaakiabg2da9iabbEha3jabb+gaVjabbkhaYjabbsgaKjabbccaGiabbggaHjabbsha0jabbccaGiabbchaWjabb+gaVjabbohaZjabbMgaPjabbsha0jabbMgaPjabb+gaVjabb6gaUjabbccaGiabdMgaPbqaaiabbggaHjabb6gaUjabbsgaKjabbccaGiabd6gaUjabg2da9iabb6gaUjabbwha1jabb2gaTjabbkgaIjabbwgaLjabbkhaYjabbccaGiabb+gaVjabbAgaMjabbccaGiabbohaZjabbwgaLjabbghaXjabbwha1jabbwgaLjabb6gaUjabbogaJjabbwgaLjabbohaZbaacaWLjaGaaCzcamaabmaabaGaeG4mamdacaGLOaGaayzkaaaaaa@80A0@

The selection of the length of the *k*-mers to analyze involves a trade-off between the statistical power gained by the large numbers that can be counted for short words and the more complete motif description that can be obtained from longer words. The size of our data sets ranges from a few hundred to a few thousand sequences, making tetramers a reasonable size choice.

### Motif finding

The DSE region spanning 80 nt downstream of the cleavage site was examined by the Gibbs Recursive Sampler [30], MEME [31] and Improbizer [29]. From each species, 500 3'-processing site sequences were randomly selected without replacement. As a position independent control, 500 sequences were generated from a species specific trained 0^*th *^order model and run in parallel. Several prelimiary runs were performed for each program to define optimal settings. At least 10 independent production runs were performed for each dataset. The Gibbs Recursive Sampler extracted 3 variable length motifs using command line options "-E 3 -W 0 -F -t -n -r -i 200 OS 200 -d 1,5,10,2,5,10,3,5,10". Motifs described in the "optimal" output section were used in order to maximize the number of example motifs tabulated. The MEME program was run a beowulf cluster using options "-dna -mod oops -nmotifs 3 -text -p52 -maxsize 1000000". Improbizer runs used options "numMotifs = 3 background = l maxOcc = l" and for additional control runs the "controlRun = on" parameter was set. Motif sequence information was gathered from all three programs via perl script and used to make sequence logo [32] images with the WebLogo script [43]. Custom perl scripts were written to collect and graph positioning from the Gibbs Recursive Sampler, Improbizer, and MEME output files.

### Multiple Alignment of CstF-64 proteins

Species specific CstF-64 protein sequences were downloaded from NCBI, UCSC and Ensembl where available. CstF-64 GenBank accessions used are as follows *H. sapiens *[GenBank:AAP88780.1], *M. musculus *[GenBank:NP_573459.1], *G. gallus *[GenBank:NP_001006433.1], *D. rerio *[GenBank:AAH65442.1], *D. melanogaster *[GenBank:AAO45216.1], and *A. gambiae *[GenBank:EAA05544.1]. Additional CstF-64 sequences for *C. familiaris, R. norvegicus, T. rubripes *and *C. elegans *were generated via tblastn [44] of closely-related available sequences (*e.g., M. musculus *used as a query of *R. norvegicus *of genomic or EST sequences) followed by assembly with CAP3 [45]. Multiple alignment of CstF-64 was accomplished using ClustalX version 1.82 [46] and displayed with UCSF Chimera sequence viewer [47].

## Authors' contributions

JS collected and pre-processed all data. JS performed the analysis with existing software packages. JS and JHG designed the novel statistical analysis and wrote the necessary software. JS, KWH and JHG analyzed and interpreted data and wrote the manuscript. JS and JHG created the supplemental web site.

## Figures and Tables

**Table 1:** Upstream (relative to the 3'-processing site) usage of AAUAAA, AWUAAA (W = A or U) or Delta 1 (AAUAAA plus all single base substitution variations).

**Organism**	**Min EST**	**Sites**	**% AAUAAA**	**Count**	**% AWUAAA**	**Count**	**% Delta 1**	**Count**
***H. sapiens***	10	10060	69.9	7032	86.0	8654	97.5	9806
***C. familiaris***	2	980	70.9	694	86.5	847	95.6	936
***R. norvegicus***	5	9329	69.7	6501	85.3	7958	97.3	9074
***M. musculus***	5	8543	70.9	6053	86.0	7342	97.0	8288
***G. gallus***	2	3056	70.0	2140	85.2	2604	96.0	2932
***T. rubripes***	1	1427	69.9	997	88.3	1259	96.6	1377
***D. rerio***	3	2585	77.9	2012	91.1	2355	98.1	2535
***A. gambiae***	1	1693	61.8	1046	71.7	537	88.9	1505
***D. melanogaster***	2	902	61.7	556	73.8	665	97.0	874
***C. elegans***	1	1003	51.0	511	71.6	717	87.7	879

**Table 2:** Invertebrate amino acid changes for residues previously identified as critical in either helix C to *β*-sheet [22] or RNA to *β*-sheet [23] interactions.

**Organism**	**F**_45_	**R**_46_	**L**_47_	**T**_53_	**Y**_59_	**C**_62_	**N**_91_	**N**_99_	**L**_101_	**L**_104_
***A. gambiae***	L	K	L	S	F	C	N	S	M	L
***D. melanogaster***	L	K	L	S	Y	C	N	S	M	L
***C. elegans***	I	K	M	T	Y	I	S	N	F	S
